# Neurofilament light chain as a biomarker for diagnosis of multiple sclerosis

**DOI:** 10.17179/excli2021-3973

**Published:** 2021-08-16

**Authors:** Ebrahim Kouchaki, Fatemeh Dashti, Seyed Mohammad Ali Mirazimi, Zahra Alirezaei, Seyed Hamed Jafari, Michael R. Hamblin, Hamed Mirzaei

**Affiliations:** 1 MS Fellowship, Department of Neurology, School of Medicine, Physiology Research Center, Kashan University of Medical Sciences, Kashan, Iran; 2 School of Medicine, Kashan University of Medical Sciences, Kashan, Iran; 3 Student Research Committee, Kashan University of Medical Sciences, Kashan, Iran; 4 Department of Medical Physics, School of Medicine, Isfahan University of Medical Sciences, Isfahan, Iran; 5 Paramedical School, Bushehr University of Medical Sciences, Bushehr, Iran; 6 Medical Imaging Research Center, Shiraz University of Medical Sciences, Shiraz, Iran; 7 School of Medicine, Shiraz University of Medical Sciences, Shiraz, Iran; 8 Laser Research Centre, Faculty of Health Science, University of Johannesburg, Doornfontein 2028, South Africa; 9 Research Center for Biochemistry and Nutrition in Metabolic Diseases, Institute for Basic Sciences, Kashan University of Medical Sciences, Kashan, IR, Iran

**Keywords:** multiple sclerosis, neurofilament light chain, diagnosis

## Abstract

The treatments for multiple sclerosis (MS) have improved over the past 25 years, but now the main question for physicians is deciding who should receive treatment, for how long, and when to switch to other options. These decisions are typically based on treatment tolerance and a reasonable expectation of long-term efficacy. A significant unmet need is the lack of accurate laboratory measurements for diagnosis, and monitoring of treatment response, including deterioration and disease progression. There are few validated biomarkers for MS, and in practice, physicians employ two biomarkers discovered fifty years ago for MS diagnosis, often in combination with MRI scans. These biomarkers are intrathecal IgG and oligoclonal bands in the CSF (cerebrospinal fluid). Neurofilament light chain (NfL) is a relatively new biomarker for MS diagnosis and follow up. Neurofilaments are neuron-specific cytoskeleton proteins that can be measured in various body compartments. NfL is a new biomarker for MS that can be measured in serum samples, but this still needs further study to specify the laboratory cut-off values in clinical practice. In the present review we discuss the evidence for NfL as a reliable biomarker for the early detection and management of MS. Moreover, we highlight the correlation between MRI and NfL, and ask whether they can be combined.

## Introduction

Multiple sclerosis (MS) is a neuroinflamamatory and neurodegenerative disease, which has different manifestations, and it is also difficult to predict its prognosis. Some patients may not need any treatment at all, but in others, the disease progresses rapidly and does not respond to standard therapy (Sartori et al., 2017[93]). Recently some new treatments have been introduced with higher efficacy, despite showing somewhat more toxicity compared to previous treatments (Rae-Grant et al., 2018[84]). As these increasingly successful treatments have become more widely available, reliable early detection and staging of disease becomes much more important (Giovannoni, 2018[38]). If physicians have more information about the prognosis and can distinguish patients with a poor prognosis, they may be able to improve the overall outcomes.

At present there is no validated biomarker for the prognosis of MS or to help clinicians choose the best therapy. Many clinicians use annual MRI examinations to evaluate the disease progression and prognosis, but MRI has limited sensitivity and is also expensive (Rovira et al., 2015[90]; Igra et al., 2017[52]).

Neurofilament light chain (NfL) is located within the myelinated neurons of the central nervous system (CNS). NfL is released when neuronal cells are damaged, and was discovered to fact as a biomarker in the CSF, reflecting the disease state in many neurodegenerative and traumatic disorders (Teunissen and Khalil, 2012[102]). Improvements have been made in methods for accurate quantification of NfL in serum samples, which were shown to correlate with CSF levels, and also to be correlated with MRI findings and clinical disease symptoms (Kuhle et al., 2016[57]). Recent studies have revealed that NfL could be used as a biomarker to predict the MS disease activity, severity, prognosis, and also to monitor treatment response (Kuhle et al., 2015[59]; Disanto et al., 2016[23], 2017[24]; Varhaug et al., 2018[111]; Thebault et al., 2019[104]). Herein, we summarize the evidence for NfL as a reliable biomarker for the early detection and disease management in MS. Moreover, we highlight the correlation between MRI and NfL measurements, and ask whether they can be combined.

## Neurofilament Light Chain Structure and Function

**Figure 1:**
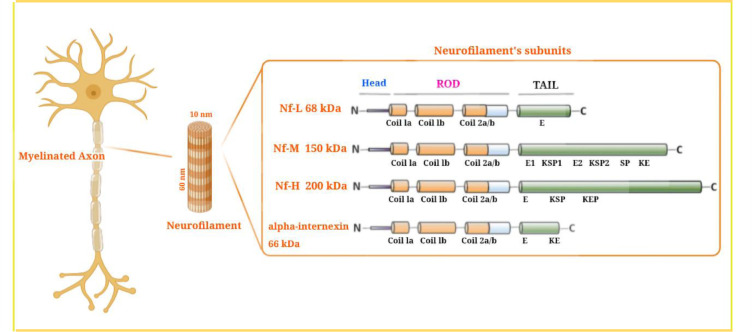
Neurofilament structure and neurofilament subunits. Neurofilaments (Nfs) are abundantly expressed in large caliber myelinated axons. Nfs are cylindrical shaped with a 10 nm diameter, lying between actin (6 nm) and myosin (15 nm). They provide a stable structural framework in axons, and allow a faster conduction velocity. Nfs are classified as intermediate filaments (iF) within the central nervous system (CNS). Components of Nfs include α-int (α-internexin), NfH (neurofilament heavy chain), NfL (neurofilament light chain), and NfM (neurofilament medium chain). All neurofilaments have three regions: an α-a conserved helical rod domain, along with variable amino-terminal and carboxy-terminal domains. The number of these components governs the variable molecular weight, so that NfH has the highest molecular weight. In the NFH tail, there is an E-segment (glutamic-acid-rich), multiple KSP repeats (lysine-serine-proline) that are phosphorylated, and a KeP segment (lysine-glutamic acid-proline). The NfM tail contains E1 and E2 segments (two glutamic-acid-rich segments), two KSP repeat segments, a SP (serine-proline), and a Ke segment (lysine-glutamic acid). The NfL tail contains an E-segment, a Ke-segment, and an α-int tail. This figure is adapted from Gaetani et al. (2019a).

Neurofilament proteins are heteropolymeric proteins that contain a mixture of components, including light (NfL), medium (NfM), or heavy (NfH) chains, which are characterized by their molecular mass (Figure 1(Fig. 1); Reference in Figure 1: Gaetani et al., 2019[31]). (Fuchs and Cleveland, 1998[30]). Recently, two other proteins, peripherin (within the peripheral nervous system) and α-internexin (in the central nervous system) have been identified as neurofilament heteropolymers (Yuan et al., 2006[117], 2012[119]). Neurofilament proteins possess an amino-terminal domain, a variable carboxy-terminal domain, and a central helical rod domain that researchers believe regulates the oligomer formation (Ching et al., 1998[17]). The C-terminal domains control the differences in phosphorylation and molecular mass between the subunits. Tetrameric neurofilament proteins are distributed bidirectionally along the axons, after synthesis and assembly of the microtubular apparatus within the neuronal cells, thereby creating a constantly overlapping sequence that runs parallel to the axons. After their formation, they are stable over the long term, even for years (Millecamps et al., 2007[70]). Neurofilaments are most abundant in mature myelinated axons (Fliegner and Liem, 1991[29]). They play a structural role in axonal cells where they help to resist external forces, govern the diameter of the axons, indirectly moderate conduction velocity, and act as a connecting track for organelles and other proteins (Yuan et al., 2017[118]). They can also play a dynamic function beyond their primary static structural function as a unique pool of synaptic neurofilament proteins (Bragina and Conti, 2018[13]). The phosphorylation of neurofilaments could be involved in long-term memory (Hashimoto et al., 2000[47]), and the stability of the NMDA receptor is based on a synaptic scaffold of neurofilament proteins. 

## Detection of Neurofilament Light Chain

Laboratory methods for diagnosing neurodegenerative diseases are based on the measurement of soluble biomarkers. As discussed above, Nfs are divided into three groups based on their tail molecular weight (Teunissen and Khalil, 2012[102]). When neuronal cells or their axonal membranes are damaged, Nfs are released into the IF (interstitial fluid), CSF, and blood, so the levels of Nf in the blood can be used for monitoring disease progress, treatment response, or drug toxicity. Blood Nf levels could potentially play a role in selecting treatment options. 

Many studies have shown that in different kinds of neurological diseases, the NfL levels can be measured in the CSF (Deisenhammer et al., 2009[22]; Teunissen et al., 2009[101]). However, clinicians cannot readily use CSF samples because they must be obtained by an invasive method, but instead they can easily use serial blood samples. Reliable quantification of NFL in blood would be a significant advance in the use of biomarkers for neurodegenerative disease diagnosis. The NF-light ELISA kit (UmanDiagnostics AB, Umeå, Sweden) is a commercially available method to measure NfL, and is very specific, but at present the manufacturer does not recommend using blood samples. UmanDiagnostics NFL-ELISA uses mAB47:3 and mAB2:1 (two non-competing monoclonal antibodies) to quantify soluble NfL (Norgren et al., 2002[72]). Two new methods for NfL measurement have been described, including electrochemiluminescence (ECL)-based assays, and a single-molecule array technology (Simoa, Quanterix, Billerica, MA, USA). ECL was developed for NfL measurement in serum and is highly sensitive, has a broad dynamic range, with the additional advantage of requiring only a small sample. Simoa is a format for digital immunoassay and could improve the sensitivity in the future (Rissin et al., 2010[86]; Gaiottino et al., 2013[35]; Wilson et al., 2016[115]).

## Neurofilament Light Chain as a Biomarker in the Diagnosis of CNS Disorders

**Figure 2:**
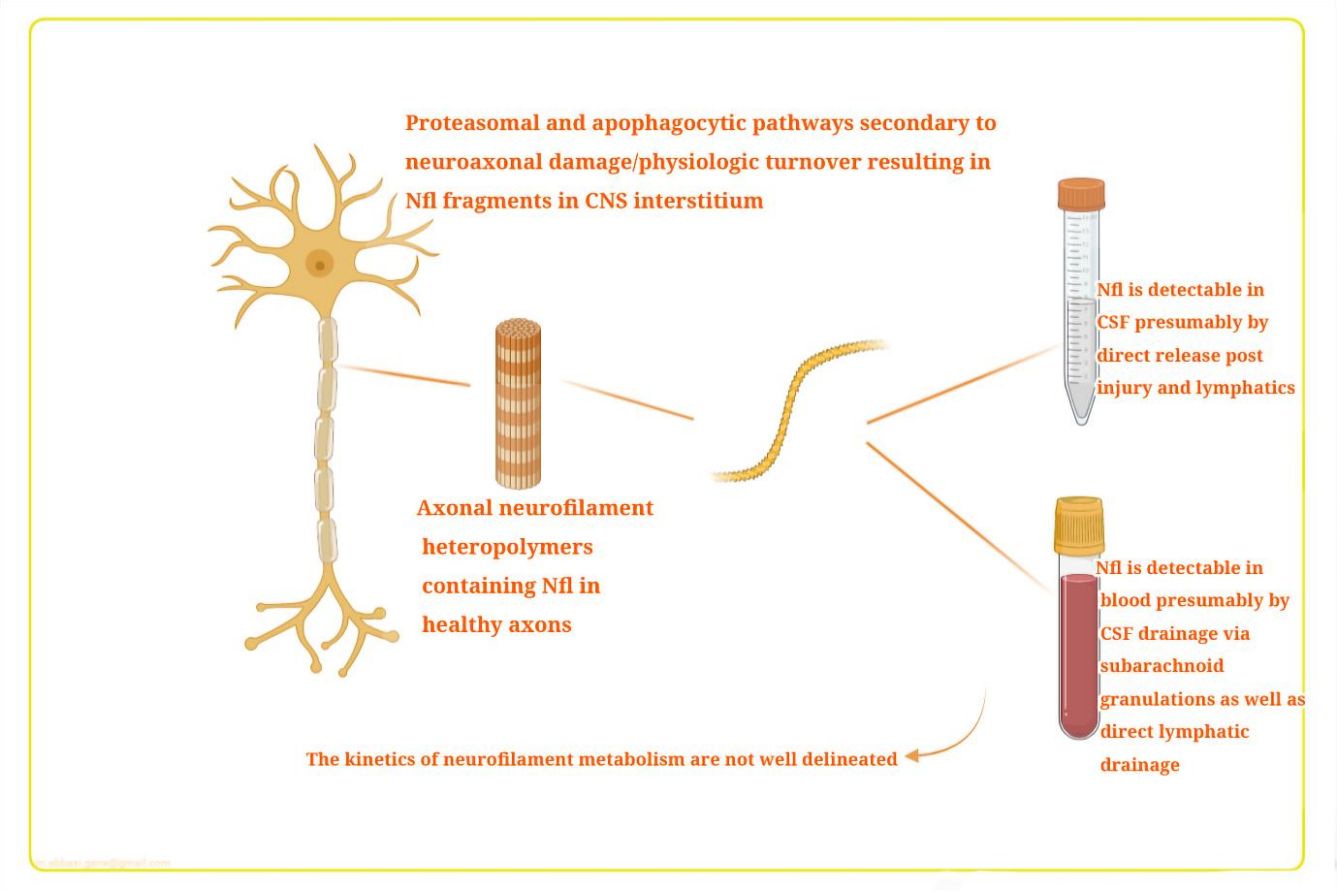
Neurofilament light chain (NfL) pathophysiology in CSF and blood. Mechanisms of transfer from damaged CNS tissue into the blood. This figure is adapted from Thebault et al. (2020).

The NfL levels in the CSF and blood are increased when the CNS is damaged (Figure 2(Fig. 2); Reference in Figure 2: Thebault et al., 2020[103]). It is proposed that when neurons are destroyed, the NfL is released as a result of a combination of two physiologic pathways, ubiquitin-mediated proteasomal hydrolysis and phagocytosis of apoptotic cells (Bomont, 2016[12]). Partially degraded neurofilaments are released into the blood and CSF via different routes, and also undergo lymphatic drainage into the perivascular and subarachnoid spaces, where they are drained into the blood and CSF via arachnoid granulations (Carare et al., 2008[16]; Gafson et al., 2020[34]). There have been good correlations reported between blood NfL levels and CSF levels with R-values (ranging from 0.7 to 8) (Thebault et al., 2019[104]). 

NfL levels have been investigated in a number of neurodegenerative disorders, including multiple sclerosis (MS) (Disanto et al., 2017[24]), Alzheimer's disease (AD) (Mattsson et al., 2017[68]), atypical Parkinsonian disorder (APD), Parkinson's disease (PD) (Hansson et al., 2017[45]), amyotrophic lateral sclerosis (ALS) (Lu et al., 2015[63]), frontotemporal dementia (FTD) (Rohrer et al., 2016[89]), and Huntington's disease (HD) (Constantinescu et al., 2009[19]; Byrne et al., 2017[14]). The NfL levels in the blood and CSF were increased in disorders characterized by proteopathic brain lesions, including tauopathy, β-amyloidosis, and α-synucleinopathy as shown in animal studies (Bacioglu et al., 2016[6]). NfL levels in plasma were higher in some disorders such as, β-amyloid autosomal dominant dementia, and MCI (mild cognitive impairment) (Mattsson et al., 2017[68]). There has been no study comparing NfL levels in Parkinson disease dementia (PDD) versus non-demented PD patients (PDND), but new research has revealed that physicians can use blood NfL levels to distinguish APD from PD. NfL levels were increased in progressive supranuclear palsy (PSP), multiple system atrophy (MSA), and CBS (cortical basal syndrome) compared to PD patients and healthy controls (Hansson et al., 2017[45]).

Yung-Shuan Lin et al. evaluated 283 patients. Of these, 119 patients had AD, 56 patients had mild cognitive impairment (MCI), 26 patients had non-demented PD (PDND), and the other 23 patients had Parkinson's disease dementia (PDD), while the 59 controls were cognitively healthy (Lin et al., 2018[62]). All participants underwent a battery of neuropsychological tests. NfL levels were measured twice in plasma using NF-light assay (NFLA) and the SIMOA method. Plasma NfL was significantly increased in the AD group, compared with, PDD, PDND, MCI, and the control groups. In AD and PD, high plasma NfL levels were linked to poor cognitive function, but not to motor symptoms. Plasma NfL was significantly higher in the PDD group, compared with the PDND group. Therefore, plasma NfL could be a biomarker for cognitive impairment in AD and PD, with greater accuracy for AD (Lin et al., 2018[62]).

The NfL levels in CSF have been measured using the UmanDiagnostics NF-light R ELISA kit, as a prognostic, pharmacodynamic, and predictive biomarker in a variety of neurodegenerative disorders (Bacioglu et al., 2016[6]). The use of blood samples is considered preferable to CSF samples, but the ELISA method has relatively low sensitivity, and because NfL concentrations are lower in blood, these evaluations have been traditionally been limited to CSF samples. However, the Single Molecule Array (Simoa™) platform is a new method with higher sensitivity so it can be reliably used to assess NfL levels in blood. A few studies have used the more sensitive electrochemiluminescence (ECL) and Simoa technologies to evaluate serum NfL levels (Kuhle et al., 2016[58]; Siller et al., 2019[98]). These studies have shown that NfL measurements in the blood, as well as the CSF, can be used as biomarkers for diseases including MS (Disanto et al., 2017[24]; Siller et al., 2019[98]).

Hendricks et al. carried out a study using bovine and human samples for NfL calibration, using the Simoa NF-light R Advantage Kit in plasma and serum, as well as CSF (Hendricks et al., 2019[48]). Plasma samples and CSF samples were obtained from 112 MS patients and were evaluated with reference to bovine and human calibrations. Their results showed that there was a meaningful correlation between CSF and blood NfL levels in MS, and between bovine and human NfL calibrations, with a conversion factor of approximately 5:1 (Hendricks et al., 2019[48]).

## Neurofilament Light Chain as a Biomarker for Diagnosis of Multiple Sclerosis

Multiple sclerosis (MS) is an autoimmune disease, where the immune system attacks the myelin sheath that surrounds and protects the neuronal cells and axons. Studies have shown that the presence of intrathecal IgG molecules has been correlated with poor prognosis and worsening disability in MS (Gasperi et al., 2019[36]). The immune response in patients who have MS is characterized by the presence of intrathecal autoantibodies against a variety of antigens (extractable nuclear antigens and oligoclonal bands) in the CSF (Jarius et al., 2017[54]). These autoantibodies can be detected in the laboratory using the MRZ reaction against three neurotropic viruses (M measles; R rubella; Z varicella zoster virus) in the CSF of MS patients (Felgenhauer and Reiber, 1992[27]; Reiber et al., 1998[85]). The MRZ reaction has been shown to be extremely specific for primary progressive MS, as well as for relapsing-remitting MS, making it a good option for diagnosis in cases with suspected primary progressive MS (Abdelhak et al., 2017[1]; Hottenrott et al., 2017[50]).

Tilman Robinson and colleagues evaluated the relationship between the clinical findings, presence of axonal damage or glial activity, and B cell biomarkers in samples from patients who were measles virus, rubella virus and varicella zoster virus (MRZR) negative or MRZR positive (Robinson et al., 2020[87]). In their multicenter cohort study, 81 PPMS patients who were either MRZR-positive or MRZR-negative underwent measurements of CXCL-13 (chemokine CXC ligand 13), BAFF (B cell-activating factor), sBCMA (soluble B cell maturation antigen), CHI3L1 (chitinase-3-like protein 1) in the CSF using ELISA kits and sTACI (transmembrane activator and CAML interactor). NfL and GFAP (glial fibrillary acidic protein) were measured in CSF and serum using SIMOA technology. The results demonstrated that in MRZR-positive patients, the NfL levels in the CSF were higher than in the MRZR-negative patients. There were no differences in clinical features between the MRZR-positive and MRZR-negative patients, as well as CSF markers such as CXCL-13, sTACI, sBCMA, BAFF, GFAP, and CHI3L1, or serum concentration of NfL and GFAP. However there was a significant correlation between NfL concentrations in serum and CSF, as well as a positive correlation between intrathecal IgG and sTACI or sBCMA. Furthermore, they suggested that in MRZR-positive PPMS patients with more axonal damage, the presence of IgG was correlated with sTACI and sBCMA (Robinson et al., 2020[87]).

With recent improvements in immunoassay platform sensitivity, researchers and clinicians have been able to measure a variety of biomarkers in the serum of MS patients. In particular, sNfL has been associated with disease progression, prognosis, and response to treatment, and also with MRI markers of disease (Disanto et al., 2017[24]; Barro et al., 2018[7]; Cantó et al., 2019[15]; Jakimovski et al., 2019[53]; Kuhle et al., 2019[60]; Siller et al., 2019[98]). Serum GFAP has been correlated with disease severity, and it is higher in PMS (progressive MS) compared to RRMS. s-GFAP is an intermediate astocyte cytoskeletal protein (Abdelhak et al., 2018[3], 2019[2]; Högel et al., 2020[49]).

A cohort study by Xavier Ayrignac and colleagues evaluated sNFL and s-GFAP levels in 129 MS patients (Ayrignac et al., 2020[5]). 111 out of 129 patients had PPMS while the other 18 had RRMS. They showed that the sNFL and s-GFAP levels were correlated with each other. Furthermore, both biomarkers were higher in PPMS compared to RRMS. However, analysis revealed that the s-GFAP measurements were significantly different. Their results showed that the s-GFAP levels correlated with the white matter lesion load by MRI, but had an inverse correlation with white matter and grey matter volume (Ayrignac et al., 2020[5]).

Electronic database findings suggest that patients with MS required medical attention more often in the five years prior to the first demyelinating event, compared to control individuals, and were more likely than controls to report a variety of psychiatric signs and symptoms to their general physician up to ten years before their MS diagnosis (Wijnands et al., 2017[114]; Disanto et al., 2018[25]).

An analysis was carried out to determine whether the serum NfL level, a possible biomarker of continuing neuroaxonal degeneration, was higher in the years leading up to and around the time of clinical onset, and thus could be a direct predictor of the MS prodromal process (Khalil et al., 2018[56]).

Bjornevik et al. evaluated the correlation between sNfL levels and clinical MS onset. They carried out a nested case-control study to determine whether sNFL could be a diagnostic biomarker to predict the clinical onset of MS (Bjornevik et al., 2020[11]). The samples were collected from US military staff who had serum samples stored in the US Department of Defence Serum Repository between 2000 to 2011, but the evaluation was done in 2018-2019. They chose 60 cases from 245 previously identified patients, and randomly identified control individuals that were matched by sex, age, date of sample collection, and race/ethnicity. SerumNfL levels were measured by the highly sensitive SIMOA technique. Their results showed that increased sNfL levels within a presymptomatic individual were linked to an increased risk of developing MS in the future. Significantly, the time of clinical onset was associated with sNfL levels, which were found to be elevated 6 years before clinical MS commenced. In conclusion, MS shows a prodromal period during which axonal damage gradually occurs, therefore if physicians recognized this phase, they could help patients avoid disability by starting treatment earlier (Bjornevik et al., 2020[11]).

Manouchehrinia and colleagues evaluated the correlation between plasma NfL levels and MS disease progression (Manouchehrinia et al., 2020[66]). In their study, they randomly selected 1026 patients based on sex and age from 4385 patients who were diagnosed with MS and, measured the pNfL levels with a highly sensitive SIMOA kit. Age-stratified pNfL levels above the 99^th^, 95^th^, and 80^th^ percentiles were correlated with the severity of EDSS (Expanded Disability Status Scale). In conclusion, they found that high pNfL levels were correlated with a sustained worsening of disability, so pNfL could be used as a clinical prognostic biomarker to predict the risk of developing permanent disability after the early stage of MS (Manouchehrinia et al., 2020[66]).

sNfL levels were correlated with T2 lesion load by MRI and gadolinium enhanced lesions in RRMS patients, as well as correlating with spinal and brain atrophy (Disanto et al., 2017[24]; Barro et al., 2018[7]; Cantó et al., 2019[15]; Siller et al., 2019[98]). More research, particularly large-scale real-world cohort and longitudinal studies are needed to translate the use of sNFL into a common biomarker used in clinics.

Recent research has revealed that sNfL can be used as a biomarker in the early stages of MS. Recent findings have suggested that sNfL might serve as a biomarker in the very early stages of MS, namely in patients with clinically isolated syndrome (Siller et al., 2019[98]), pediatric MS (Wong et al., 2019[116]), or in the pre-symptomatic stages of MS (Bjornevik et al., 2020[11]). Physicians have used McDonald's criteria for MS diagnosis, which were first presented in 2001, and underwent many revisions (last McDonald version in 2017; Thompson et al., 2018[105]). The major criteria were based on dissemination in space (DIS), and dissemination in time (DIT).

The McDonald criteria allow physicians to diagnose RRMS at the first clinical presentation, and can help to distinguish CIS, in which the chronic nature of MS symptoms is not yet apparent, by the lack of either DIS or DIT (Thompson et al., 2018[105]). The definition of DIT was significantly changed in the last revision of McDonald's criteria: (1) presence of a symptomatic lesion which is enhanced by Gd; (2) presence of OCB (oligoclonal bands) in the CSF (Filippi et al., 2016[28]). The last revision attempted to diagnose MS earlier in order to begin disease-modifying therapy (DMT), to improve the prognosis and lessen long-term morbidity and mortality. An analysis revealed that the 2017 McDonald criteria aided in the earlier diagnosis of RRMS compared to the 2010 McDonald criteria, particularly by using OCB in the DIT criteria (Beesley et al., 2018[8]; Schwenkenbecher et al., 2018[95]; McNicholas et al., 2019[69]).

Stefan Bittner and colleagues evaluated the use of sNfL as a biomarker for defining the chronicity of MS and detecting axonal damage in their multicenter (22 centers) prospective longitudinal observational cohort study (Bittner et al., 2020[10]). Their study included 814 patients diagnosed with either RRMS or CIS, who were admitted between August 2010 and November 2015. The sNfL levels were measured by SIMOA, and the clinical symptoms and MRI scans were evaluated. 445 out of 814 patients were diagnosed with RRMS, and the other 369 patients were diagnosed with CIS (using the 2010 criteria). After reclassification with the 2017 criteria, they found that sNfL levels were lower in CIS (2017 criteria) compared to RRMS (2017 criteria) patients, and the Gd-positive T2 lesions correlated with sNfL levels at baseline to predict future clinical relapse. Longitudinal shifts in sNfL levels reflected the escalation in therapy required over this time span. Using sNfL in disease assessment improved its diagnostic accuracy. The sNfL levels were correlated with disease prognosis, and by measuring longitudinally, improved therapeutic decisions (Bittner et al., 2020[10]). Table 1(Tab. 1) (References in Table 1: Arrambide et al., 2016[4]; Ayrignac et al., 2020[5]; Barro et al., 2018[7]; Bhan et al., 2018[9]; Bittner et al., 2020[10]; Bjornevik et al., 2020[11]; Dalla Costa et al., 2019[20]; Disanto et al., 2016[23], 2017[24]; Engel et al., 2019[26]; Gaetani et al., 2019[32][33]; Gil-Perotin et al., 2019[37]; Gunnarsson et al., 2011[40]; Haghighi et al., 2004[41]; Håkansson et al., 2017[44], 2018[43]; Hendricks et al., 2019[48]; Jakimovski et al., 2019[53]; Kuhle et al., 2015[59], 2016[58], 2017[61], 2019[60]; Lycke et al., 1998[64]; Malmeström et al., 2003[65]; Manouchehrinia et al., 2020[66]; Modvig et al., 2015[71]; Norgren et al., 2003[73]; Novakova et al., 2017[74][75][77], 2018[76]; Olesen et al., 2019[78]; Panek et al., 2020[79]; Piehl et al., 2018[82]; Quintana et al., 2018[83]; Robinson et al., 2020[87]; Sejbaek et al., 2019[96]; Sellebjerg et al., 2019[97]; Siller et al., 2019[98]; Thebault et al., 2019[104]; Tortorella et al., 2018[106]; van der Vuurst de Vries et al., 2019[110]; Watanabe et al., 2019[112]; Wong et al., 2019[116]; Zhang et al., 2007[120]) lists various studies that have measured NfL levels in MS patients. 

## Combination of Neurofilament Light Chain measurements with Imaging Techniques in Diagnosis of Multiple Sclerosis

The combination of immunohistochemistry techniques and MRI scans have led to the detection of neuroaxonal injury at earlier stages in many neurological disorders, including RRMS and CIS (Trapp et al., 1998[107]; Haider et al., 2016[42]; Gracien et al., 2017[39]). NfL was the most commonly investigated biomarker, and a correlation between CSF NfL levels and neuroaxonal injury was found during the entire course of MS, with values of NfL being 10 times higher than the normal upper limit during clinical acute relapses (Malmeström et al., 2003[65]). Levels of NfL in CSF and serum are strongly correlated with each other, and the serum NfL values in patients with RRMS are significantly higher than in healthy individuals (Kuhle et al., 2016[58], Disanto et al., 2017[24]). 

Siller et al. performed a study to evaluate the correlation between serum levels of NfL during chronic or acute onset neuroaxonal injury in the early stages of RRMS (Siller et al., 2019[98]). NfL values in serum were measured by the SIMOA technique in 74 patients recently diagnosed with RRMS or CIS (Siller et al., 2019[98]). Highly sensitive 3 T MRI scanning was carried out at the beginning of the study and during subsequent follow-up visits in 42 patients for between 6 to 37 months. The baseline NfL levels in serum were correlated with the volume of brain lesions in T2 MRI, but were not correlated with patient age, EDSS score, or other measured MRI variables. However, patients with increased baseline serum levels of NfL had a more rapid progression in the reduced volume of brain tissue and a higher number of brain lesions in T2 MRI. Contrast-enhancing lesions showed a positive correlation with NfL values in serum. Disease modifying therapy resulted in a marked reduction of NfL levels in serum. Increased levels of serum NfL at baseline were associated with higher risk of developing brain atrophy in the following 2 years, suggesting that serum NfL may be used as a new indicator of early neuroaxonal injury in MS patients (Siller et al., 2019[98]).

Baseline NfL values could predict future disease activity and progression within the study groups. In the FREEDOMS study, the NfL levels were divided into low and high NfL levels reflecting the probability of developing new or expanding lesions in T2 MRI, incremental loss of brain tissue, and more frequent relapses over 2 years, regardless of treatment administration. Studies measuring NfL levels in serum (Piehl et al., 2018[82]) as well as CSF (Arrambide et al., 2016[4]; Petzold et al., 2016[81]) confirmed the prognostic value to predict future brain atrophy. This was further validated by measuring deterioration of the spinal cord in another observational study (Barro et al., 2018[7]).

Kuhle and colleagues evaluated NfL levels in blood as a biomarker of past, current, and future disease activity, and brain atrophy in RRMS patients, and to evaluate the response to treatment (Kuhle et al., 2019[60]). They measured blood NfL levels in 35 healthy individuals and 589 patients with RRMS, taken from the FREEDOMS study, and phase 3 studies of interferon [IFN]-β-1a, fingolimod vs placebo, and the TRANSFORMS study. They further evaluated the prognostic value of NfL to predict MRI results and clinical outcomes. Levels of NfL (pg/mL) were significantly higher in MS patients compared to healthy controls, and were closely linked to the number of T2 lesions and T1 gadolinium-enhanced lesions. Higher levels of NFL at the beginning of the study were significantly (CI=95 %) associated with a higher number of expanding or new lesions in T2 MRI, increased frequency of relapse, reduced volume of brain tissue, and higher probability of disability progression. Treatment with fingolimod was associated with a significant reduction in blood levels of NfL over a 6-month-period, and this effect remained unchanged during the whole study. Levels of NfL in blood correlated well with disease activity measures, neuroaxonal injury by MRI lesions, and clinical features of patients at their presentation. Moreover, blood levels of NfL may predict the final outcome of MS patients (Kuhle et al., 2019[60]). As mentioned above, increased NfL levels can be detected in the CSF or blood of patients with neurologic disorders causing axonal damage, such as MS (Lycke et al., 1998[64]; Malmeström et al., 2003[65]). Evaluation of pathologic abnormalities in the grey matter using MRI has been suggested to evaluate the clinical significance of NfL measurements. However, evaluating brain volume loss via imaging approaches is difficult, and employing retrospective assessments of patient brain volume in clinical settings remains unreliable (Wattjes et al., 2015[113]). According to previous studies, NfL levels can be used to detect neuroaxonal injury in conjunction with MRI (Lycke et al., 1998; Malmeström et al., 2003[65]).

Damasceno et al. measured NfL levels in patients with RMS and PMS, and investigated the correlation between clinical features and MRI parameters with NfL levels. Forty-seven patients with MS underwent 3T MRI evaluation and a LP (lumbar puncture) to obtain CSF (Damasceno et al., 2019[21]). NfL concentrations were detected by ELISA, and a multi-variable regression analysis was run to evaluate the relationship between NfL levels, MRI variables, and clinical features. The CSF NfL levels were linked to previous disease activity in patients with RMS, and to contrast-enhancing lesions and the volume of cortical lesions. Multivariable analysis showed a significant correlation between clinical relapse within the last 12 months and presence of cortical lesions with the NfL levels. In patients with PMS, the volume of contrast-attenuating lesions in T1 MRI was only correlated to the NfL levels. Although contrast-enhancing lesions and NfL levels were related to each other, no correlation was found with previous disease activity. They suggested taking into account the history of clinical disease activity when evaluating the relationship between MRI and NfL levels (Damasceno et al., 2019[21]).

In a study by Costa et al., the prognostic value of serum NfL in patients presenting with CIS (according to McDonald 2017 criteria) and CDMS (clinically definite MS) was evaluated (Dalla Costa et al., 2019[20]). This retrospective cohort study included patients presenting to the Neurology Department between 2000-2015, with a first episode of demyelination-related events. Baseline NfL levels in serum and CSF, as well as clinical symptoms and MRI variables were obtained. Out of 222 patients participating in the study (mean follow-up of approximately 100.6 months), 45 patients (20 %) developed into CDMS and 141 patients (63.5 %) progressed to McDonald 2017 criteria MS within two years. NfL levels in serum (median of 22.0 and an interquartile range of 11.6-40.4 pg/mL) were significantly higher among patients with previous recent relapses, and were closely linked with an increased number of contrast-enhancing lesions in T2 baseline MRI. The NfL levels in serum were predictive of McDonald 2017 MS and CDMS development. Low levels and extremely low levels of serum NfL were associated with a 2-fold and 3-fold reduced risk of disease progression, respectively. This finding did not change after adjusting for other prognostic factors in MS patients such as gadolinium enhanced lesions, presence of OCB in the CSF, and an increased number of lesions in T2 baseline MRI. Baseline NfL levels were correlated with disability, however their increase over time was not associated with increased disability. NfL levels in serum had the capability to predict the evolution of CIS into MS. High and increasing NfL levels reflect serious underlying inflammatory processes during the course of the disease, whereas steady NfL levels may reflect the presence of underlying chronic inflammation and neurodegeneration (Dalla Costa et al., 2019[20]).

SyMRI® (synthetic MRI) can be used to evaluate the brain volume as a marker of BPF (brain parenchymal function) using easy-to-apply methodology, with relatively short scan and post-processing times (Vågberg et al., 2013[109]). Standard MRI scanning is limited by technical constraints, physiological heterogeneity, and challenges in determining cut-off thresholds for following the depletion of brain volume (Sastre-Garriga et al., 2017[94]). On the other hand, measuring the BPF is not a suitable technique to evaluate changes in MS patients over follow-up periods of months to years (Rocca et al., 2017[88]). However, measuring changes in the NfL levels in the CSF over time can be valuable in clinical settings, suggesting it could be a suitable candidate to be included in the NEDA (no evidence of disease activity) criteria, although cut-off thresholds must be determined.

Håkansson and colleagues investigated the relationship between serum and CSF NfL levels, and brain atrophy in RRMS and CIS patients, and the subsequent disease progression (Håkansson et al., 2018[43]). A panel of neuroinflammatory and neurodegenerative markers were evaluated in CSF samples from 41 patients with RRMS or CIS in a prospective cohort study, and in CSF samples of 22 healthy individuals. Serum NfL was measured by the SIMOA technique. In this study, NEDA-3 criteria as well as brain tissue volume (BPF by SyMRI®) were measured during four years follow-up of the patients. Serum and CSF NfL levels were both correlated with disease progression, however CSF NfL levels showed a more significant correlation with NEDA-3 criteria, brain atrophy, and new lesions in T2 MRI. The CHI3L1 (chitinase-3-like protein 1) level in the CSF was strongly correlated with brain atrophy, and the CSF levels of CXCL1, MMP-9, CCL22, CXCL13, and CXCL10 were correlated with the development of new lesions in T2 MRI. CSF NfL levels were a better predictor of disease activity compared to serum NfL levels (Håkansson et al., 2018[43]).

Although the relationship between NfL levels in serum and MRI evaluations and clinical outcomes of patients has been widely investigated (Disanto et al., 2017[24]; Barro et al., 2018[7]; Kuhle et al., 2019[60]), only a few studies have investigated the correlation between disruption of the BBB (blood brain barrier) integrity and serum NfL levels (Kalm et al., 2017[55]; Novakova et al., 2017[74]). Moreover, other risk factors for MS development, including HLA DRB1 (human leukocyte antigen DRB1 locus gene) status (Huizinga et al., 2012[51]), immune response against EBV, and 25-hydroxycholecalciferol (25-hydroxy vitamin D3) (Sandberg et al., 2016[92]; Smolders et al., 2020[99]) have not been investigated in relation to NfL levels and the development of axonal injury.

In a study by Uher et al., the relationship between levels of NfL and markers of inflammation in the CNS, along with measurements of the BBB integrity were investigated in MS patients, at their presentation with an initial demyelinating event (Uher et al., 2021[108]). CSF and blood samples were acquired from 142 patients diagnosed with MS (based on McDonald 2017) who received no treatment after the onset of clinical symptoms. Levels of albumin, NfL, IgG, and IgM were evaluated in blood and CSF samples. Albumin was measured in the CSF as a marker of BBB integrity. Flow cytometry was used to measure the number of immune cells present in the CSF. Other risk factors for the development of MS, including HLA DRB1, 25-hydroxy vitamin D3, and antibodies against EBV were also evaluated. Higher levels of NfL in the serum were correlated with increased albumin, CD80+CD19+ double positive cells, and CD80+ cell numbers in the CSF. NfL levels in serum were also correlated with a higher number of gadolinium-enhancing lesions in T2 MRI. CSF albumin was not associated with the other evaluated MS markers. In addition, serum NfL levels were correlated with levels of anti-EBV IgG against VCA (viral capsid antigen) (Uher et al., 2021[108]).

NfL baseline levels have been measured prior to starting disease-modifying treatments. Furthermore, NfL measurements were able to predict the severity of prolonged disability and progression of neuro-degeneration (Piehl et al., 2018[82]). The CLIMB study found an association between NfL levels in serum at the beginning of each year, the average annual NfL concentrations, MRI-based outcomes, and disease progression and weakness over a 10-year-follow-up period (Chitnis et al., 2018[18]). The EPIC study showed that NfL levels in serum at baseline could predict the loss of brain volume over the next 2-10 years (Cantó et al., 2019[15]).

Häring and colleagues compared the prognostic value of measuring NfL levels in plasma over 12 and 24 months, with a single measurement of NfL levels in patients presenting with RRMS. They further addressed the combination with different MRI and clinical variables (Häring et al., 2020[46]). This study analyzed the data from Phase 3 clinical trials. The FREEDOMS study (FTY720 Research Evaluating Effects of Daily Oral therapy in MS) which continually administered fingolimod for 24-months in RRMS patients. The TRANSFORMS study (Trial Assessing Injectable Interferon vs FTY720 Oral in RRMS) over 12 months, and LONGTERMS (their long-term extension). In the Häring analysis patients were categorized into two distinct groups on the basis of their baseline NfL levels, or the mean NfL values measured longitudinally at 12 and 24 months, to compare brain atrophy and disease progression. These groups were low (<30 pg/mL, n = 164) and high (≥30 pg/mL, n = 110). A higher baseline NfL level predicted an increased risk of developing an EDSS score ≥4 at an earlier time point, as well as increased brain volume loss within 120 months. High NfL levels measured over 24 months, also predicted an EDSS score >4, worsening of disability within 6-months, and a 20 % reduction in the Timed 25-Foot Walk Test. The values of area under the ROC curve were increased when NfL levels were combined with MRI-based and clinical assessments (Häring et al., 2020[46]).

It has been shown that CSF-NfL levels have the ability to predict the development of new lesions in MRI, clinical relapses, progression of disability, and response to treatment in MS patients (Lycke et al., 1998[64]; Teunissen et al., 2009[101]; Håkansson et al., 2017[44]). Other studies have investigated the prognostic value of NfL or NhL concentrations in the CSF with overall survival of the patients (Salzer et al., 2010[91]; Martínez et al., 2015[67]; Soelberg Sorensen and Sellebjerg, 2016[100]) as well as MRI variables (Petzold, 2015[80]).

In a study by Chitins et al. the prognostic value of yearly measurements of NfL levels in serum of MS patients to predict 10-year MRI and clinical outcome was evaluated (Chitnis et al., 2018[18]). MS patients referred to the Center for Comprehensive Longitudinal Investigations at Brigham and Women's Hospital (CLIMB) study within 5-years from the onset of their disease were included, and they underwent yearly blood sampling for at least 10 years (n = 122). Serum NfL concentrations were measured by SIMOA. The volume of T2 contrast-enhancing brain lesions (T2LV) and BPF was measured during the final year of this study using 3T MRI, and an automated algorithm. In this study, the yearly average NfL levels were inversely correlated with BPF at year 10. The average NfL levels during the first 5 years of the study showed a better correlation than the NfL levels measured during the last 5 years of the study. About 15-20 % of changes in BPF and T2LV could be correlated to NfL values during the first 5 years. The average values of NfL did not show a significant correlation with EDSS scores at year 10, or with Timed 25-Foot Walk or the Symbol Digit Modalities Test (a measure of concentration and decision making). They concluded that serum NfL levels assessed over the initial years after disease onset, may serve as prognostic tool to predict brain atrophy and MRI lesions for up to 10 years in MS patients (Chitnis et al., 2018[18]).

## Conclusions

Over recent years, measurements of NfL concentrations in blood and CSF have been shown to provide a valuable diagnostic ability to detect neuroaxonal injuries in different neurological disorders. Although alterations in NfL levels in biologic fluids may not be specific for any pre-determined neurologic disorder, their measurement over time may reflect disease activity and response to treatment. Even though there is an expanding list of studies supporting the clinical utility of NfL measurements, additional studies are required to confirm the analytical and clinical reliability, prior to its widespread use in different clinical settings. One limitation to NfL measurements, is the absence of a validated method for comparing NfL levels in the blood and CSF in different studies. Better standardization and more inter-laboratory studies may help to confirm the reproducibility of these assays and their validity when performed in different laboratories. Additionally, the normal levels of NfL for each specific age group, need to be determined when employing NfL for each individual patient. As a result, studies involving larger numbers of healthy controls must be performed to produce a set of data for normal age ranges. NfL levels in blood, allows monitoring of the effects produced by administration of various treatments on the integrity of neuronal tissues. Moreover, NfL may be used as a predictor of final outcome, especially in clinical trials investigating new treatments (e.g. phase IIA and IIB studies), where the optimum drug dosage needs to be established. Taken together, additional studies are required to confirm the correlations between longitudinal changes in NfL levels (particularly in blood) and neuroimaging results and clinical signs in different neurological disorders. Finally, measuring changes in the NfL levels in the CSF over time can be valuable in clinical settings, suggesting it could be a suitable candidate to be included in the NEDA (no evidence of disease activity) criteria, although cut-off thresholds must be determined.

## Notes

Ebrahim Kouchaki, Fatemeh Dashti and Seyed Mohammad Ali Mirazimi contributed equally as first authors.

Seyed Hamed Jafari, Michael R. Hamblin (Laser Research Centre, Faculty of Health Science, University of Johannesburg, Doornfontein 2028, South Africa; E-mail: Hamblin.lab@gmail.com) and Hamed Mirzaei (Research Center for Biochemistry and Nutrition in Metabolic Diseases, Institute for Basic Sciences, Kashan University of Medical Sciences, Kashan, I.R. Iran; Tel: +98-31-55540022, Fax: +98-31-55540022, E-mail: mirzaei-h@kaums.ac.ir, h.mirzaei2002@gmail.com) contributed equally as corresponding authors.

## Figures and Tables

**Table 1:**
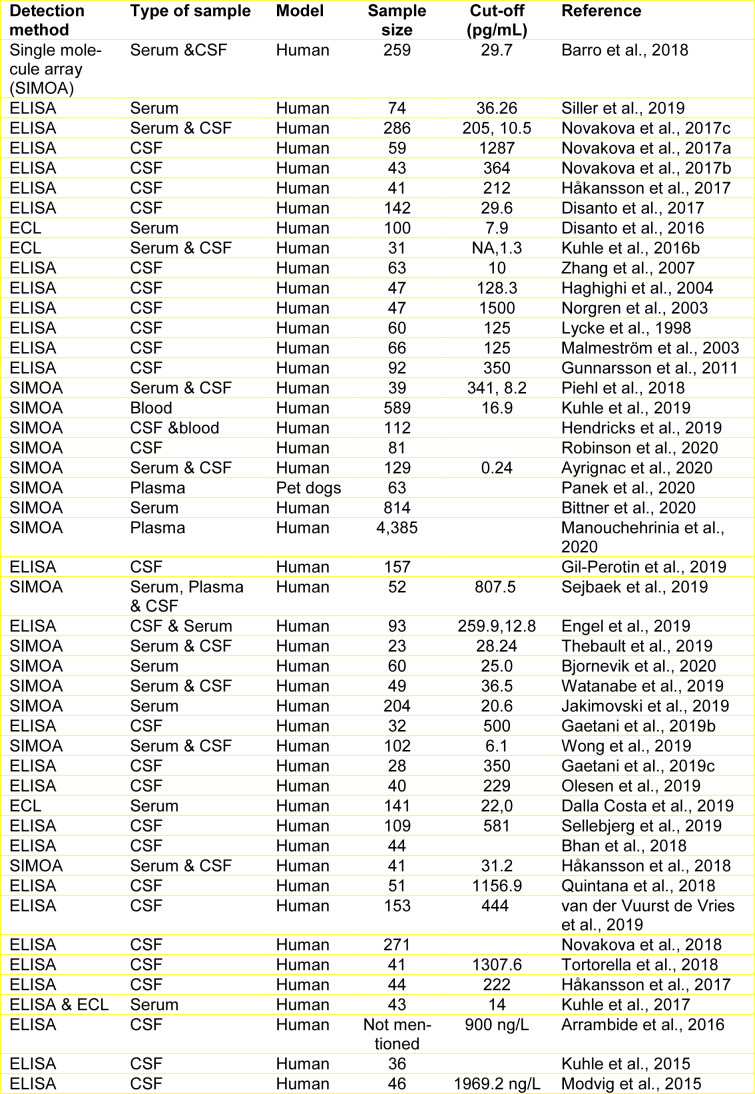
Various clinical studies on the levels of NfL in MS patients
